# Four Puzzles of Reputation-Based Cooperation: Content, Process, Honesty, and Structure

**DOI:** 10.1007/s12110-021-09419-3

**Published:** 2021-12-28

**Authors:** Francesca Giardini, Daniel Balliet, Eleanor A. Power, Szabolcs Számadó, Károly Takács

**Affiliations:** 1 Department of Sociology, University of Groningen, Grote Rozenstraat, 31 - 9712 TG Groningen, Netherlands; 2 Department of Experimental and Applied Social Psychology, VU Amsterdam, Boechorststraat 1, 1081 BT Amsterdam, The Netherlands; 3 London School of Economics and Political Science, Department of Methodology, Houghton Street, WC2A 2AE London, UK; 4 Department of Sociology and Communication, Budapest University of Technology and Economics, Egry J. u. 1. Floor 7, 1111 Budapest, Hungary; 5 The Institute for Analytical Sociology, Linköping University, 601 74 Norrköping, Sweden; 6 Centre for Social Sciences, CSS-RECENS, Tóth Kálmán u. 4, 1097 Budapest, Hungary

**Keywords:** Gossip, Reputation, Cooperation, Evolution, Honesty

## Abstract

Research in various disciplines has highlighted that humans are uniquely able to solve the problem of cooperation through the informal mechanisms of reputation and gossip. Reputation coordinates the evaluative judgments of individuals about one another. Direct observation of actions and communication are the essential routes that are used to establish and update reputations. In large groups, where opportunities for direct observation are limited, gossip becomes an important channel to share individual perceptions and evaluations of others that can be used to condition cooperative action. Although reputation and gossip might consequently support large-scale human cooperation, four puzzles need to be resolved to understand the operation of reputation-based mechanisms. First, we need empirical evidence of the processes and content that form reputations and how this may vary cross-culturally. Second, we lack an understanding of how reputation is determined from the muddle of imperfect, biased inputs people receive. Third, coordination between individuals is only possible if reputation sharing and signaling is to a large extent reliable and valid. Communication, however, is not necessarily honest and reliable, so theoretical and empirical work is needed to understand how gossip and reputation can effectively promote cooperation despite the circulation of dishonest gossip. Fourth, reputation is not constructed in a social vacuum; hence we need a better understanding of the way in which the structure of interactions affects the efficiency of gossip for establishing reputations and fostering cooperation.

Cooperation—undertaking costly actions that benefit others—has been heralded as one of the most impressive human qualities. Humans are especially adept at cooperating under conditions where many other species fail—that is, with genetically unrelated individuals who share limited potential for future interactions (Gächter & Herrmann, 2009). This kind of cooperation is difficult to explain, but in recent decades work in several different disciplines has emphasized reputation as a solution (Barclay, 2012; Suzuki & Akiyama, 2005; Wu et al., 2016). Broadly, reputation is a set of evaluations that are held about the qualities of an individual. Formal models (Leimar & Hammerstein, 2001; Nowak & Sigmund, 1998a; Panchanathan & Boyd, 2004; Santos et al., 2018) have focused on reputations as proxy of simple and observable behaviors, such as donating (or not) to another individual. Reputations can be based on knowledge of others’ past cooperative behavior, either through previous interactions, direct observation, or exchange of information about third parties, which is called gossip (Barkow, 1992; Dores Cruz et al., 2021; Emler, 2019).

Building a reputation as a cooperator can result in direct benefits (Roberts, 1998), such as being selected for a long-term partnership, or indirect benefits (Bliege Bird et al., 2001; Milinski, 2016), such as receiving help from others who have not been the recipient of one’s previous cooperation. Thus, reputations carry the potential to attract new partners, and they further enable cooperators to assort and avoid costly interactions with those who will not cooperate. Reputation can therefore be seen as an instrument for self-organization in society. Evidence from different kinds of small, close-knit communities (Boehm, 2019; Brenneis, 1984; Ellickson, 1994; Greif, 1989) as well as from larger groups and organizations (Ellwardt et al., 2012; Kniffin & Wilson, 2005; Wittek & Wielers, 1998; Yoeli et al., 2013) consistently shows that reputation can be a powerful instrument of social coordination.

Research on reputation and cooperation, however, has largely overlooked the role of gossip, while portraying reputation as an equivalent of direct observation of a simple action (Hess & Hagen, 2019; Számadó et al., 2021). Theoretical accounts and experimental studies tend to describe cooperative decisions as being based on simple actions (to cooperate or not) which are widely observable. Whenever this information is transmitted, it is usually understood to be a reliable equivalent of direct observation. Although models of indirect reciprocity and competitive altruism have greatly contributed to our understanding of the workings of reputations (Leimar & Hammerstein, 2001; Nowak & Sigmund, 1998b; Ohtsuki & Iwasa, 2004, 2006; Roberts, 2020), we argue that a new approach is needed to tackle several aspects of reputation that have yet to receive theoretical and empirical attention. We highlight four open questions that can be used as cornerstones for future developments of a new interdisciplinary theoretical framework of reputation-based cooperation. For the sake of clarity, we present them as four distinct issues, but they are deeply entangled.

Before turning to these four puzzles about the substance of reputational judgements, the process of reputation formation, the reliability of gossip, and the structure of interactions, we first introduce indirect reciprocity and competitive altruism as foundational models describing the interplay among reputation, gossip, and cooperation. We conclude with some general remarks about the importance of addressing these issues.

## The Foundational Models

Theories of reputation-based cooperation posit that agents monitor and evaluate others’ actions and then condition cooperation on these evaluations (Barclay, 2016; Nowak & Sigmund, 2005; Roberts, 1998). In models of “indirect reciprocity” (Alexander, 1987; Boyd & Richerson, 1989; Nowak & Sigmund, 1998a; Panchanathan & Boyd, 2004), individuals decide to cooperate (or not) with others, and this is reflected in the “image score,” which will affect whether third parties cooperate with them (or not) in a future encounter (Leimar & Hammerstein, 2001; Nowak & Sigmund, 1998a). An image score is a summary of past actions that is visible to every other player and is updated after each interaction. In Nowak and Sigmund’s original model, donors can use the recipient’s image score to decide whether to help. Indirect reciprocity can explain cooperation in contexts where (1) the population is large and reencounters are unlikely, (2) interactions are one-shot, (3) individuals have no control over whom they interact with (i.e., random partner matching), and even where (4) the updating of reputational scores is subject to some random noise. These models, however, assume that individuals have (an infinite) cognitive capacity to maintain a record of the past actions and reputations of others (Basu et al., 2009; Mullins et al., 2013); this knowledge is publicly shared; and most typically also that this knowledge is received in a reliable and honest form.

In models of “competitive altruism” (Barclay, 2016; Barclay & Barker, 2020; Roberts, 1998), individuals choose partners to engage in mutually beneficial interactions, competing in a “biological market” (Noë & Hammerstein, 1995) to establish more cooperative reputations that will make them more appealing partners. This competition is a core assumption of these models and implies that individuals (1) are incentivized to increase their “market value”; (2) actively compare between options, weighing the relative generosity (or other valuable trait) of partners when making the decision to interact; and (3) determine their own generosity based on their knowledge of a potential partner’s market value (and their own). Although the early models emphasized the cost of “sampling” the market and getting information (Noë & Hammerstein, 1994, 1995), it is often taken for granted that individuals can readily assess the relative quality of all parties.

Indirect reciprocity and competitive altruism are both extremely influential explanations of cooperation that have helped highlight the powerful role that reputation can play in fostering cooperation. As our brief summaries make clear, however, they also rest on a set of simplifying assumptions. These assumptions make modeling tractable, but they also mean that the models diverge substantially from the complex process of building, assessing, and using reputations in real-world contexts. For example, Macfarlan and colleagues (Macfarlan et al., 2013) looked in detail at the alignment (or not) between cooperative acts and prosocial reputations in Dominica, finding that the simple models of indirect reciprocity did not capture the actual process of reputational formation.

A set of questions needs to be asked to have a fuller understanding of real-world reputational systems. For each of these questions we will suggest potential avenues for future research and relevant interdisciplinary collaborations, with the aim to advance the current understanding of reputation-based cooperation and to contribute to the development of an interdisciplinary theory. First, it should be clear that people do not have perfect knowledge of everyone’s past actions. What is the actual substance of people’s reputations, and how does this vary cross-culturally? Second, there is gap in our theorizing about reputation management: How does people’s awareness of the self-interested motivations of others to manage their own reputations shape how they assess and integrate the many (potentially biased) reputational inputs they receive? A third missing piece in the theory relates to the information on which reputations are built. If reputations are built largely on gossip, then there is a substantial risk of inaccurate or biased inputs, not the perfect records the models assume. How do individuals gain sufficiently reliable information on the actions and attributes of others so they can make informed decisions about their future relations? Fourth, people do not interact at random. Humans are embedded in complex, multifaceted, clustered networks. How does the structure of social interactions shape how reputations are formed? Here, we outline why these questions matter, the partial answers we can give to date, and what could be done to answer them more fully. It is beyond the scope of this review to formulate a new theory of reputation-based cooperation, but addressing these four challenges can be regarded as a first step toward theory building.

## What Is the Substance of Reputational Judgments, and How Do They Vary across Contexts?

What is it that reputational assessments broadly comprise? Across a variety of fields, social scientists have suggested that we are concerned both with evaluating people’s qualities and also with how those people are likely to relate to *us* in particular. This has been variably called competence and morality (Wojciszke, 1994), competence and warmth (Fiske et al., 2007), quality and intentions (Roberts, 2015), ability and willingness to confer benefits (Barclay, 2016), and capital and character (Barker et al., 2019). The social psychological literature clearly documents that we form not only judgments of others’ reputational qualities, but also expectations of others’ behavior, and whether they are worthy partners for future engagement (Yao et al., 2014).

Studies on the content of gossip provide some insight into the substance of our reputational assessments. Much of the time spent in conversation with others is passed discussing social topics, such as personal relationships (Dunbar et al., 1997; Emler, 1994), highlighting our interest in the intentions and dispositions of others. A recent study of gossip in a Dutch community sampled instances of participants sending and receiving gossip across 10 days and found that the majority of gossip could be used to evaluate the target of gossip according to trustworthiness, warmth, competence, and dominance (Dores Cruz et al., 2021). It is further notable that gossip is often evaluative, not necessarily negative, and lab studies show that people tend to gossip about defectors (Samu et al., 2020; Sommerfeld et al., 2007) and norm violations. Field studies observing different groups, such as California cattle ranchers (Ellickson, 1994) and college rowing teams (Kniffin & Wilson, 2005), have also found that gossip is often about individuals who fail to live up to the group’s expectations (McAndrew, 2019).

So, people’s actions (and gossip about that action) are used to formulate reputational assessments that span a number of different domains, including trustworthiness, prosociality, competence, dominance, and norm compliance (Molho et al., 2020; Shank et al., 2019). How might the relative importance of these attributes vary within and between different sociocultural settings? Garfield et al. (2021) present the results of an exploratory analysis of ethnographic texts on reputation from 153 cultures, revealing substantial variation in reputational domains cross-culturally. This suggests that different reputational qualities may be particularly valued and valuable in different contexts (see also (Romano et al., 2021). Macfarlan and Lyle (2015), for example, find common reputational domains but also differences between Dominica and Peru. Their results suggest that reputations for economic competency affected cooperation across more social contexts than prosocial reputations, a finding that can be largely explained by the specific socioeconomic context of goods production. Among the Tsimane of Bolivia, male leaders were found to be rated as more trustworthy and physically dominant, but not consistently as more generous or knowledgeable (von Rueden et al., 2014), suggesting that certain reputational domains are valued among leaders, and others, not. Among the Chabu in Ethiopia, in contrast, learning and intelligence was the strongest predictor of male leadership, with physical strength not being related to leadership status (Garfield et al., 2019). Since these two groups are both forager-horticulturalists, differences in subsistence strategies cannot explain the differently valued reputational domains. Cross-cultural comparative work on the effect of status on male reproductive success has similarly found that the effects of physical formidability, hunting skill, material wealth, and political influence did not vary across societies based on the type of subsistence (von Rueden & Jaeggi, 2016). Notably, although leaders among the Chabu are central in organizing collective activities, such practices are reportedly rare among the Tsimane (Gurven & Winking, 2008); such differences in the importance of collective action may help to explain the different reputational qualities that are valued in leaders (Garfield et al., 2019).

Beyond the question of the various sources of reputational information is the question of how any such inputs will be judged. This can be further specified as input depending on the cooperation context, i.e., the payoff structure and design elements, and input in terms of the receiver’s assumption about the sender’s strategic motivations to influence us. Experimental work shows that even in very abstract situations such as a dictator game, the evaluation of an action depends on the choice set (Bardsley, 2008; List, 2007), and this is even more likely outside a controlled laboratory experiment. This aspect has been systematically overlooked by current theories of reputation, which tend to focus only on few abstract structures (donor-receiver interaction, collective dilemmas). The recently developed Cooperation Databank (Spadaro et al., 2020)—a database consisting of a standardized annotation of the entire history of research on human cooperation in social dilemmas—could be used to evaluate how variation across abstract cooperation contexts (e.g., the degree of conflicting interests, repeated interaction, anonymity) affects how different kinds of partner reputations (e.g., trustworthy, dominant) relate to decisions to cooperate. Such work could contribute to the development of a more comprehensive theory of reputation-based cooperation in which scope conditions are clearly outlined.

Of course, different reputational qualities may be valued in different sorts of people, suggesting that there will be different reputational domains *within* societies as well. The qualities that are found in leaders, for example, may not necessarily be the reputational domains that everyone works to pursue; only relatively few may aspire to positions of prominence within a community (Power & Ready, 2018). Unfortunately, much of the cross-cultural work on status and leadership has focused exclusively on men; recent work (Post & Macfarlan, 2020) suggests that women’s reputations are often less well defined in the ethnographic record, but they appear to become more salient in matrilineal societies. Among the Tsimane (von Rueden et al., 2018) and the Chabu (Garfield & Hagen, 2020), women’s reputational domains are indeed different from men’s, with less emphasis on dominance and knowledge. Gaining a reputation for generosity has been noted as beneficial across a wide range of settings (e.g., Gurven et al., 2000; Lyle & Smith, 2014), and this may be a valuable reputational domain for women (Power & Ready, 2018).

While this work hints at some patterns guiding the content of valued reputational domains within and between societies, more can be done to characterize and explain variation in reputation cross-culturally. Ethnographic observational work is needed to provide evidence about mechanisms and processes of reputation and gossip in different ecologies, institutional settings, and cultures. Through long-term observational work, it may be possible to identify what sorts of actions, by what sorts of individuals, lead to reputations in particular domains (e.g., Power, 2017). There is also a need for cross-cultural studies of reputation formation, in which key dimensions, their relative importance, and how these dimensions are integrated can be further explored. Content analysis or topic modeling of audio, textual, and online social media records could also help define topics of gossip and domains of reputational concerns in a range of social settings (Robbins & Karan, 2020). Interdisciplinary collaborations between psychologists, sociologists, and anthropologists could lead to the design of vignette studies and surveys with large-scale cross-cultural samples to identify whether there are any universals in reputation formation and management, and, where there is variation, what might account for it (King & Cowlishaw, 2007). Overall, future research is needed to better clarify the general domains and dimensions of reputational concern—e.g., trustworthiness, competency, morality, status—on which conditional judgments are made for short-term cooperation and long-term commitment, and how these vary between individuals and between societies.

## How Are Reputations Shaped?

If the quality of interactions with others could be summarized and expressed on the basis of one or a few objective criteria, as happens in theoretical models of reputation-based cooperation and in online markets (Dellarocas, 2003; Diekmann et al., 2014; Tadelis, 2016), identifying trustworthy partners would be easy. Whereas simple models of image score, in which cooperative or uncooperative actions have a direct effect on someone’s desirability as a partner, may be sufficient for other species (e.g., cleaner fish; Bshary & Grutter, 2006), humans’ capacity for language and our highly elaborated theory of mind make the process of interpreting communicative and reputation-building acts particularly complex (Dunbar, 1998, 2004). It remains unclear how humans draw upon and integrate the many muddled inputs they have at their disposal to formulate the reputational assessments outlined above.

A person’s inferences about another’s reputation may be built on the basis of many potential factors, including potentially subtle cues, contextual features, prior knowledge, and social information (Fiske, 1993). When formulating a reputational assessment, how much weight do individuals give to their direct interactions versus their observations of others, or the circulating social information they hear (i.e., gossip)? Models of social learning provide some information on when and how these various inputs will be drawn upon (e.g., Kendal et al., 2018), but how those various inputs will then be weighted and integrated also needs to be assessed (e.g., King & Cowlishaw, 2007). Furthermore, social psychology has long chronicled the biases that exist in how we process social information, suggesting that the reliance on such inputs may warp reputational assessments in important ways (Dumas et al., 2021; Hills, 2018; Ross & Nisbett, 1980). Most of the formal models of reputation-based cooperation skirt this issue by assuming that reputation can track behavior with the same accuracy as direct experience, letting agents incorporate this new information as if it were gleaned from direct, personal experience (Nowak & Sigmund, 2005).

Managing the impressions one makes on others is a cornerstone of social life (Baumeister, 1982; Goffman, 1958). According to theories of impression management, our actions are partly driven by the intention to elicit particular (positive) reputation judgments from our partners (Goffman, 1958). Early psychological work shows that how someone behaves depends as much on who they are as on with whom they interact (Kenny et al., 2001). For instance, while some signals might be aimed at general aggrandizement, others may be more targeted, subtle signals of cooperative intent toward particular others (Bliege Bird & Power, 2015; Bliege Bird et al., 2018; Schroeder et al., 2019). Recent research indicates that from about 5 years of age, children engage in self-promotional strategies (for a review, see Engelmann & Rapp, 2018) and that they try to maintain a positive reputation (Fu et al., 2016).

Cognizant of these efforts at impression management, human decision-makers try to understand the intentions behind others’ actions, not simply evaluating the action itself but also considering that people actively work to manage their reputations and how others perceive them. Humans exercise “epistemic vigilance” (Sperber et al., 2010), being particularly attuned to the risk of potential misinformation. This means that someone’s actions or the information about them can be discounted if the receiver assumes that the actor has an interest in manipulating the receivers’ beliefs and influencing their actions. The complexity of this process is considerably amplified when gossip is considered. Language provides humans with the ability to expand their reputation management strategies and benefit from unique opportunities both to improve their reputations (Giardini et al., 2019, 2021) and to destroy others’ (Besnier, 2009). With gossip, recipients need to evaluate not only the significance of the reputational information about the third party being discussed, but also how the motivations and reputation of the gossiper might shape their portrayal of that third party.

Future research should investigate how individuals integrate the multifaceted reputational inputs they receive and how they manage their reputations, particularly considering that people exercise epistemic vigilance and can be extremely skeptical and selective in their judgments. Experimental work could manipulate the reputational inputs observers receive (e.g., direct interaction versus gossip, with varying levels of “noise”) to establish how observers weigh and integrate these inputs (cf. King & Cowlishaw, 2007). Vignette studies (Mathew & Boyd, 2014; Yamamoto et al., 2020) and survey work (e.g., Power, 2017; von Rueden et al., 2008) can also be used to investigate how people evaluate hypothetical others and what kind of information they seem to consider when assessing others (Peters et al., 2017).

## What Is the Role of (Dis)Honest Gossip in Reputation and Signaling?

If much of reputation is built on what people say, then there is a further question of how third-party communication—and particularly the possibility of strategic and dishonest gossip—affects the dynamics of reputation and cooperation. The widespread presence of reputation-based cooperation and the parallel existence of lying suggest that reputational systems have to be robust in the face of inaccurate information, thus raising a key question: how can reputation support cooperation when people have an incentive to use gossip to misrepresent reality?

The growing number of experiments investigating the role of gossip in the maintenance of cooperation collectively suggest that gossip can serve as a tool to promote cooperation (Milinski, 2019; Sommerfeld et al., 2007). However, most of these experiments investigate only the “threat of gossip” (Beersma & Van Kleef, 2011; Piazza & Bering, 2008; Wu et al., 2019) or implement gossip in a situation without a conflict of interest (Feinberg et al., 2012, 2014; Sommerfeld et al., 2007, 2008) in which participants have no incentive to lie.

In almost all models and experiments, gossip is assumed to be honest (Nowak & Sigmund, 1998a; Ohtsuki & Iwasa, 2004), but this is not the case in real life. Classic ethnographic accounts of gossip highlight that it can be used strategically (e.g., Paine, 1967), and people often lie or exaggerate to manipulate others, either to improve their own reputation or to damage the reputation of their competitors (Buss & Dedden, 1990; Diekmann et al., 2014; Hess & Hagen, 2006; McAndrew, 2014). Lying can be promoted by both pro-self (DePaulo et al., 1996) and prosocial motivation (Shalvi & De Dreu, 2014; for review, see Jacobsen et al., 2018). Moreover, lying can be contagious (Kocher et al., 2017; Mann et al., 2014) and can be influenced by the expected norm violations of others (Diekmann et al., 2015). In a recent experimental study, Peters and Fonseca (2020) show that participants used gossip to lie, and lies were twice as frequent under competition between gossipers. However, lies had no discernible effect on trust levels, and exaggeration lies (in which the target’s contribution is reported as higher than it was) are the product of gossipers’ attempts to actively engineer indirect reciprocity. Simulation studies investigating the effect of the veracity of gossip on reputation and cooperation report contradictory findings. A small proportion of agents who lie to influence others’ reputations will result in the collapse of cooperation (Antonioni et al., 2016; Számadó et al., 2016), or cooperation can be maintained even when gossip is not perfectly reliable (Fonseca & Peters, 2018; Giardini & Vilone, 2016). These diverging results depend on the way in which veracity is modeled: reputation systems can be robust against a fixed error rate (Giardini & Vilone, 2016), but they are vulnerable to strategic dishonesty (Számadó et al., 2016).

Signaling theory is a useful framework for understanding the conditions under which receivers can have confidence in the reliability and honesty of the messages sent by signalers (Enquist, 1985; Fudenberg & Tirole, 1991; Grafen, 1990; Maynard Smith & Harper, 2003; Skyrms, 2010; Spence, 1973). Signaling theory assumes that individuals can have an incentive to influence others for personal gain and hence give signals intended to exert that influence. The interests of signalers and receivers are often not aligned, so receivers are therefore skeptical of the signals and look for evidence of the honesty and reliability of the information conveyed. A shared insight of these models is that honest signaling is maintained by differential marginal cost (Enquist, 1985; Grafen, 1990; Spence, 1973) or by differential marginal benefits (Godfray, 1991) in such a way that the use of signals is beneficial for honest signalers, but not for potential cheaters (Bergstrom et al., 2002; Lachmann et al., 2001). Using the insight of signaling theory, we propose that future studies should focus on measuring the marginal costs and benefits of honest and dishonest gossip. For instance, the marginal cost of signals and gossip benefits can be manipulated in lab experiments (Samu et al., 2020; Számadó et al., 2020), thus contributing to theoretical models advancing current knowledge of signaling.

Understanding the conditions that must be met for skeptical observers to have reasonable confidence in the reliability and honesty of *all* forms of communication—including gossip—is crucial for any reputation-based model of cooperation because it establishes the credibility of information about an individual’s ability and willingness to cooperate. Two main theoretical and empirical advances can be put forward in order to tackle the issue of reliability and trustworthiness of gossip: first, looking at evaluations of the source, i.e., the extent to which the receiver’s knowledge of and trust in the source affects the reputational judgment about the target; and second, assessing how much and what kind of dishonesty can be tolerated until reputation systems break down. Aside from the opportunity costs of gossip, gaining a negative reputation as a gossipmonger has several disadvantages (Farley, 2011). The link between dishonesty and reputation lies at the intersection of psychology, linguistics, evolutionary biology, and computational social science. Formal (game theoretic) modeling could be used to determine the costs and benefits of dishonesty (Dellarocas, 2003), and agent-based models could provide relevant insights about the robustness of reputation systems to cheating (Quattrociocchi et al., 2009). Text analysis and natural language processing can be applied to the study of honest intent, verbal expressions of exaggerations, and controversial content as related to (dis)honest communication in natural language corpora (Newman et al., 2003; Pennebaker, 2011). Moreover, lab experiments could be used to test how individuals process and integrate multiple inputs to infer the veracity of communication.

## How Does the Structure of Social Relations Affect Reputation and Cooperation?

The costs and benefits of gossip and the effects of reputation may also depend on the structural aspects of the interaction. Theoretical models explaining the emergence and maintenance of cooperation have long shown that structured populations provide a more realistic arena for analysis than models assuming that people interact and communicate with randomly selected others (Nowak & May, 1992). Individuals tend to interact with those who are in close proximity to them or with whom they are somehow connected, and they also compare their outcomes and adapt behavioral strategies locally or through their network ties.

Network structure can play multiple roles in determining how gossip and reputation affect cooperation (Simpson et al., 2017; Takács et al., 2021). For example, certain individuals might be in a better structural position and have access to more relevant and accurate information than others (Rooks et al., 2011). These could be individuals who are in the position to broker information between different subgroups in the social network (Burt, 2005; Giardini & Wittek, 2019). Others might face divergent information, causing insecure reputations of others or variation in evaluations across the community (Macfarlan et al., 2013). Having partners in common (triadic closure) could allow an easy cross-checking of information, whereas bridging between different social groups (structural holes) could allow for accessing multiple sources of information but makes it difficult to check its accurateness (Righi & Takács, 2018). Strategic misrepresentation might be viable for individuals in certain network positions but not for others who could be more comprehensively evaluated. This is especially relevant when there is disagreement on morality and about the value of cooperation in the community (Smith & Apicella, 2020), but also in the wake of “fake news” (Shao et al., 2018; Vicario et al., 2016; Vosoughi et al., 2018), which can contribute to the spreading of false reputations.

Just as the structural position of individuals can influence their incentive to gossip honestly or dishonestly, so too does it influence the inputs they receive. What we hear from others (as well as what we observe of others) is fundamentally shaped by the set of relationships we are already embedded within (Raub & Weesie, 1990). From lab experiments using simple communication chains (Mesoudi et al., 2006) to online experiments with structured populations (Rand et al., 2011), research shows that information tends to be transformed in the process of transmission. In real-world networks of Hungarian adolescents (Kisfalusi et al., 2019), Dutch and German employees (Beersma et al., 2019; Ellwardt et al., 2012; Grosser et al., 2010; Wittek & Wielers, 1998), and rural villagers in India (Power & Ready, 2018) and Bolivia (von Rueden et al., 2019), network properties such as density, closure, transitivity, multiplex reciprocity, and centrality have been found to influence reputation.

Certain network conditions and processes could result in substantial differences in the way in which a person is perceived by different groups of individuals (Grow et al., 2016; Kisfalusi et al., 2019). Strong clustering of the network could create filter bubbles such that individual reputations of actors are largely different in distinct segments of the network. Not just initial clustering, but also the coevolution of cooperation and networks could create “echo chambers” of reputations and polarization over time (Gross & De Dreu, 2019; Melamed et al., 2020). Such reputational divergences are clearest when we consider how reputations might span group boundaries. The same acts which foster a positive reputation within the group may lead to a negative reputation with out-group members (Raihani & Power, 2021). Whether and to what extent a lack of agreement might undermine cooperation are theoretical and empirical questions. Despite the fact that reputational dynamics and within-group cooperation are affected by competition with out-groups, current reputation-based theories generally consider reputations within the boundaries of a well-mixed social group or organization and implicitly assume that observers agree in their judgment of others’ cooperative behaviors. How reputations converge or diverge over time, whether divergences can undermine cooperation, and how these cycles of segmentation and polarization can be counteracted are still open questions. Revitalized societal polarization and intergroup conflict fueled by clustered reputations create a timely challenge for interdisciplinary work.

Future research is needed to characterize the spatial and structural characteristics that shape and distort reputation formation. The way in which gossip and reputation formation are shaped in spatially constrained interactions and in networks can be tested by means of computer simulations with evolving network structure (Gross & De Dreu, 2019; Traag et al., 2011), including data-driven ones that help to calibrate and validate models. Agent-based modeling could be a useful methodology to explore the implications of different judgments within the same populations, and the extent to which this disagreement will impact the sustainability of cooperation. The results of computational models can then be used to derive hypotheses to be tested experimentally, but also to provide indications for ethnographic studies. Empirical studies should be carried out in a variety of different social and cultural contexts since we should expect significant variation in the structure and importance of social relations across contexts. This could draw on ethnographic evidence or survey work to get comprehensive information on reputation, behavior, and social ties. Psychology, sociology, anthropology, socio-physics, and computer science can collaborate to create a versatile body of knowledge on perceptions, social influences, constraints, and the actual spread of reputations.

## Conclusions

Managing environmental resources, funding charities, governing organizational networks, organizing social movements all clearly illustrate the age-old challenge of large-scale cooperation. How can cooperation be sustained in these contexts, and more broadly in human societies, despite the temptation of selfish actions? Reputation is an excellent candidate for supporting cooperation, first because it can signal cooperative attitudes and actions, second because the possession of a good reputation can lead to individual benefits, and third because it can easily spread through gossip.

In this paper, we aim to provide a multi- and interdisciplinary synthesis and call attention to four areas for future research related to reputation, gossip, and their relationship with cooperation: (1) how consensual beliefs about attributions of others are formed and how these attributes vary cross culturally; (2) how reputations are actually composed, given the muddle of inputs people have; (3) how the potential dishonesty of communication (such as gossip) affects reputations; and (4) how the structural features of social networks impact reputations and cooperation. We claim that these four areas provide the theoretical foundations for a new theory of reputation-based cooperation.

Current theoretical accounts leave crucial issues unaddressed, and we argue that a better integration of their insights can help us move forward to a more complete explanation of human cooperation. While reputation and gossip plausibly support cooperation in large groups, there are still open challenges that should be part of an interdisciplinary framework on reputation, gossip, and cooperation.
